# Dietary cadmium exposure assessment among the Chinese population

**DOI:** 10.1371/journal.pone.0177978

**Published:** 2017-05-18

**Authors:** Yan Song, Yibana Wang, Weifeng Mao, Haixia Sui, Ling Yong, Dajin Yang, Dingguo Jiang, Lei Zhang, Yunyun Gong

**Affiliations:** 1 Key Laboratory of Food Safety Risk Assessment, National Healthand Family Planning Commission of the People’s Republic of China (China National Center for Food Safety Risk Assessment), Beijing, China; 2 University of Leeds, Leeds, United Kingdom; Leibniz-Institut fur Pflanzengenetik und Kulturpflanzenforschung Gatersleben, GERMANY

## Abstract

Cadmium, a toxic heavy metal, is widely present in food. It has been reported that chronic cadmium exposure is associated with kidney disease, osteoporosis, cardiovascular disease and cancer. The aim of this study was to assess the dietary cadmium exposure and potential health risk in different age-sex groups of the Chinese population (children aged 4–11 years, young people aged 12–17 years and adults aged over 18 years), and in the southern and northern population using a semi-probabilistic method. Cadmium was detected in 228,687 food samples from 32 food categrories by graphite furnace atomic absorption spectrometry. The dietary cadmium exposures were estimated by combining the cadmium concentration data with food consumption data derived from the China National Nutrient and Health Survey 2002, and evaluated against the Provisional Tolerable Monthly Intake (PTMI) of 25 μg/kg BW/month established by the Joint FAO/WHO expert committee on food additives (JECFA). The mean dietary cadmium exposure of the general Chinese population (15.3 μg/kg BW/month) was below the PTMI. The high consumer exposures (95^th^ percentile, P95) for the general population and different sub-groups were higher than the PTMI. The dietary cadmium exposure of the southern population was apparently higher than that of the northern population. Rice was the most important contributor to cadmium exposure for Chinese people, especially those living in the southern areas of China. These findings indicated that the health risk from dietary cadmium exposure of the general Chinese people was low, but the health risk of cadmium exposure of certain sub-groups should be of concern.

## Introduction

Cadmium, a heavy metal, is ubiquitous in the environment. It makes up 0.1 ppm of the earth’s crust and occurs naturally in the environment in its inorganic form as a result of volcanic emissions and weathering of rocks. Cadmium is always associated with sphalerite. As a consequence, mining, smelting, and refining sulfidic ores of zinc can release additional cadmium into the environment. Meanwhile, because of the widespread use of cadmium in industries and agriculture, anthropogenic activities, such as waste emission, waste incineration and the use of fertilisers, can increase the contaminantion levels of cadmium in soil, water and air, and consequently in the food chain [1]. The general population is exposed to cadmium from multiple sources, including smoking, with food accounting for approximately 90% of cadmium exposure in the non-smoking population [2]. Grain and vegetables are usually the most important contributors [3–4].

Cadmium absorption after intake is relatively low in humans (3%-5%), but cadmium is efficiently retained in the kidney and liver, with a very long biological half-life ranging from 10 to 30 years [5–6]. Cadmium has no known biological function in animals and humans but mimics other divalent metals that are essential to diverse biological functions. Cadmium is primarily toxic to the kidney, especially to the proximal tubular cells where it accumulates over time and may cause a decrease in the glomerular filtration rate, and eventually renal failure. Some toxicological studies have shown that long-term dietary exposure to cadmium can cause damage to the kidney and bone [7–9].

The International Agency for Research on Cancer (IARC) has classified cadmium as a human carcinogen (Group 1) on the basis of occupational studies [10]. Although there was not enough evidence that cadmium is carcinogenic to humans exposed orally [11], some epidemiology evidence showed that cadmium may increase the risk of cancer in the lung, endometrium, bladder, prostate and breast [12–18].

To control the damage from cadmium to human health, the Joint FAO/WHO expert committee on food additives (JECFA) established a provisional tolerable weekly intake value (PTWI) of 7μg/kg BW/week in 1988[19]. However, the committee withdrew the PTWI and established a provisional tolerable monthly intake (PTMI) of 25μg/kg BW/month at its 73^rd^ meeting based on some newer evidence from epidemiological studies and considering the long half-life of cadmium [20].

In Asia, cadmium continually increased from about 43 percent of worldwide production in 1998 to about 60 percent in 2004 [21].Among the world top ten, China was the first for cadmium refinery production in 2014 [22]. In the past decades, with the rapid growth in production, environmental cadmium pollution worsened and consequently the contamination level of cadmium in foods increased. Up to now, several heavily polluted areas have been indentified, most of which lie in the main rice-producting provinces of China [23]. Rice is one of favorite foods among the Chinese population but it is also one of the agricultural plants that tend to take up cadmium from soil. The large consumption of rice contaminated by cadmium was the most important factor causing the itai-itai disease that occurred in Japan in the 1940s [24]. Chinese total diet studies revealed that the dietary cadmium exposure of Chinese adults increased dramatically from1990 to 2000 [25–27]. It is necessary to take measures to explore the overall health risk and to identify sub-populations at high risk from cadmium exposure.

However, data on the dietary cadmium intake of Chinese sub-groups are limited. In this study, a semi-probabilistic risk assessment of dietary cadmium exposure in the Chinese population including the general population, the age-sex groups and the southern and northern population was conducted by using the food cadmium concentration data collected in China from 2011 to 2015.

## Materials and methods

### 1. Food cadmium concentration data

A total of 228,687 individual food samples were collected from supermarkets, local markets and in the field during harvest time in 31 provinces, autonomous regions and municipalities of China between 2011 to 2015.The food samples were aggregated into 32 food groups including rice, wheat flour, nuts, fish, molluscs, shrimp, crab, vegetables, mushrooms, fungi, purple seaweed, kelp, meat, liver, kidney, egg, milk, fruits, juice, and tea.

Food samples were analyzed in local laboratories in 31 provinces, autonomous regions and municipalities. All laboratories used the same analysis procedures and training was provided before the analysis.

Cadmium in foods was analysed following a protocol for elemental analysis in the China National Monitoring Handbook of Food Safety and China National Food Safety Standard GB/T 5009.15–2003. The limit of detection (LOD) ranged from 0.00001 mg/kg to 0.1 mg/kg for different food groups. All the the samples with results below the LOD were assigned the value of LOD or a half of LOD depending on the recommended method by the GEMS/Food [28]. As shown in Table 1, the overall percentage of samples below LOD was 35.7%. For milk and fresh eggs, the percentage of samples below LOD was over 60%, hence the LOD value (UB, upper bound) was assigned to those non-deteactable samples. Otherwise, ½ of LOD was assigned to the non-detectable (MB, middle bound).

**Table 1:** The consumption and concentration levels of cadmium for 32 food groups in China.

Food group	Cadmium concentrations			Food consumption (g/day)
	Sample size	<LOD of the samples (%)	Mean±SD (mg/kg)	
Rice	19,786	19.0	0.062±0.128	218.6±174.5
Wheat	20,925	19.0	0.021±0.026	145.4±168.0
Other cereals	13,766	48.8	0.008±0.019	16.5±50.0
Nuts	6,325	34.5	0.029±0.062	3.8±15.7
Fish, sea	7,011	28.5	0.022±0.199	7.6±27.2
Fish, freshwater	9,564	50.3	0.007±0.026	15.5±38.9
Molluscs	4,817	8.1	0.377±1.008	1.6±11.8
Shrimp, freshwater	4,328	26.8	0.043±0.173	0.8±6.8
Shrimp, sea	3,843	25.5	0.067±0.274	1.6±10.9
Crab, freshwater	4,345	21.6	0.101±0.323	0.7±7.3
Crab, sea	3,726	11.6	0.544±1.203	0.8±8.9
Vegetable, root & stalk	18,136	28.2	0.015±0.043	34.2±60.3
Vegetable, leafy	21,479	26.8	0.021±0.100	124.1±115.7
Vegetable,cucurbits and fruit vegetable	11,178	42.1	0.008±0.020	75.6±112.9
Vegetable, legume	4,997	43.7	0.007±0.025	2.6±16.1
Mushroom, fresh	5,451	22.0	0.040±0.119	2.0±11.2
Mushroom, dry	2,294	12.4	0.366±0.928	0.5±5.0
Fungi, edible, not incl. mushroom.	1,601	20.0	0.080±0.126	1.0±7.3
Purple seaweed	531	10.7	1.034±0.947	0.5±4.5
Kelp	207	21.7	0.168±0.366	
Kelp, dry	308	13.0	0.252±0.360	
Meat, stock	9,113	49.6	0.009±0.031	64.1±72.4
Meat, poultry	2,603	53.5	0.007±0.018	12.8±35.5
Liver	5,398	14.6	0.070±0.245	1.0±7.2
Kidney	6,061	18.6	0.222±1.024	0.1±1.9
Egg, fresh	4,238	62.1	0.007±0.034	20.7±32.7
Egg, preserved	2,658	58.4	0.008±0.023	0.6±5.1
Milk, not incl. milk powder	8,522	72.6	0.003±0.011	21.0±71.3
Milk powder	4,937	66.2	0.005±0.015	0.5±6.9
Fruits	14,373	56.7	0.004±0.009	39.1±83.7
Juice	127	56.7	0.008±0.024	0.4±8.0
Tea	6,039	13.8	0.058±0.096	0.5±11.7
Total	228,687	35.7	0.049±0.322	893.1±325.6

### 2. Food consumption data

Food consumption data were obtained from the Chinese National Nutrition and Health Survey (CNNHS) of 2002. In this survey, a total of 67,608 study subjects were selected through stratified multi-stage cluster sampling from 31 provinces, autonomous regions, and municipalities in China. Food consumption data was collected with a 24-hour dietary recall method on three consecutive days. At the same time, the individual body weight was investigated. The food consumption within each food category was summed up for each investigated person in order to match it with the cadmium occurrence data of each food category. Daily consumption data in the general population for every food category are shown in Table 1.

### 3. Estimation of the cadmium intake

Daily cadmium intake was calculated by multiplying individual food category daily consumption data with the corresponding mean concentration of cadmium of the food catergory, and added up to give estimated total daily cadmium intake. A factor of 30 days was used to get a monthly exposure for each individual. An estimate was made of the total monthly amount of cadmium for each individual.

Cadmium intake was calculated according to the following formula:

$$Exp_{i}=\sum_{j=1}^{n}\frac{F_{j}\times C_{j}}{W_{i}}\times 30$$

Where *Exp*_*i*_ denoted cadmium dietary exposure of consumer *i* (μg/kg BW/month); *F*_*j*_ denoted consumption amount of consumer *i* from food category *j* (g/day); *C*_*j*_ denoted content of cadmium in food category *j* (mg/kg); *W*_*i*_ referred to body weight of consumer *i* (kg); *n* referred to the number of food categories used to derived *Exp*_*i*_ for consumer *i*.

The mean and 95^th^ percentile of exposure were obtained for the general population and each age-sex group, namely, childrent (4–11 years), young people (12–17 years), and adults (over 18 years).

### 4. Statistical analysis

All Statistical analyses were carried out using SPSS 19.0. The results were presented as means ± SD. Comparisons between two or multiple groups were carried out using *t*-test or one-way ANOVA followed by Bonferroni post hoc comparisons tests when equal variances was assumed and Dunnett’s T3 post hoc tests when equal variances assumption was not met. Statistics with *p*-values less than 0.01 were considered to be significant.

## Results

### 1. Cadmium concentrations in foods

As shown in Table 1, the mean cadmium concentration of all foods was 0.049 mg/kg. The levels of cadmium varied considerably among food items. Purple seaweed had the highest concentration of cadmium contamination, followed by sea crab,molluscs, dry mushroom, dry kelp and kidney. In cereals (including rice, wheat and other cereals), rice had the highest mean value of cadmium (0.062 mg/kg). The leafy vegetables showed the highest mean cadmium concentration of 0.021 mg/kg among all the vegetables excluding edible fungi.

### 2. Estimation of dietary cadmium exposures of the Chinese population

#### 2.1. Dietary cadmium exposures by different sub-groups

The dietary cadmium exposures for the general population and different age-sex sub-groups of Chinese population are shown in Table 2. The mean cadmium exposure of the general population was 15.3 μg/kg BW/month, which equated to 61.2% of the PTMI.

**Table 2:** Dietary cadmium exposure levels by age-sex groups.

Population groups	Number of subjects	Cadmium exposure (μg/kg BW/month)		
		Mean±SD	Median	P95
4-11yr	8252	24.2±14.2^b^^,^^c^^,^^d^^,^^e^	21.3	48.2
12-17yr,male	3199	17.9±10.4^a^^,^^c^^,^^d^^,^^e^	15.7	35.7
12-17yr,female	2763	15.7±9.5^a^^,^^b^^,^^d^^,^^e^	13.7	31.5
≥18yr,male	25221	13.8±8.1^a^^,^^b^^,^^c^	12.0	27.4
≥18yr,female	28173	13.6±8.2^a^^,^^b^^,^^c^	11.8	27.8
General population	67608	15.3±9.9	12.9	33.0

a: statistically significant difference between 4-11yr and any other age-sex group at p<0.01

b: statistically significant difference between 12-17yr (male) and any other age-sex group at p<0.01

c: statistically significant difference between 12-17yr (female) and any other age-sex group at p<0.01

d: statistically significant difference between ≥18yr (male) and any other age-sex group at p<0.01

e: tatistically significant difference between ≥18yr (female) and any other age-sex group at p<0.01.

For Chinese adults, the mean cadmium exposure was 13.7 μg/kg BW/month for both men and women, accounting for about 54.8% of the PTMI. Children exposed to cadmium significently higher than young people and adults (p<0.01), but did not exceed the PTMI of cadmium (Table 2).

Cadmium exposure in the high consumer group (P95) of the general population and sub-groups were all above the PTMI (Table 2). The exposure level of the high consumer in the general population was 33.0 μg/kg BW/month, which was about 1.3 times of the PTMI. The exposure level of the high consumer at different age-sex groups ranged from 27.4 (male adult) to 48.2 (children 4–11 years) μg/kg BW/month, which were 1.1–1.9 times of the PTMI.

**Fig 1:**
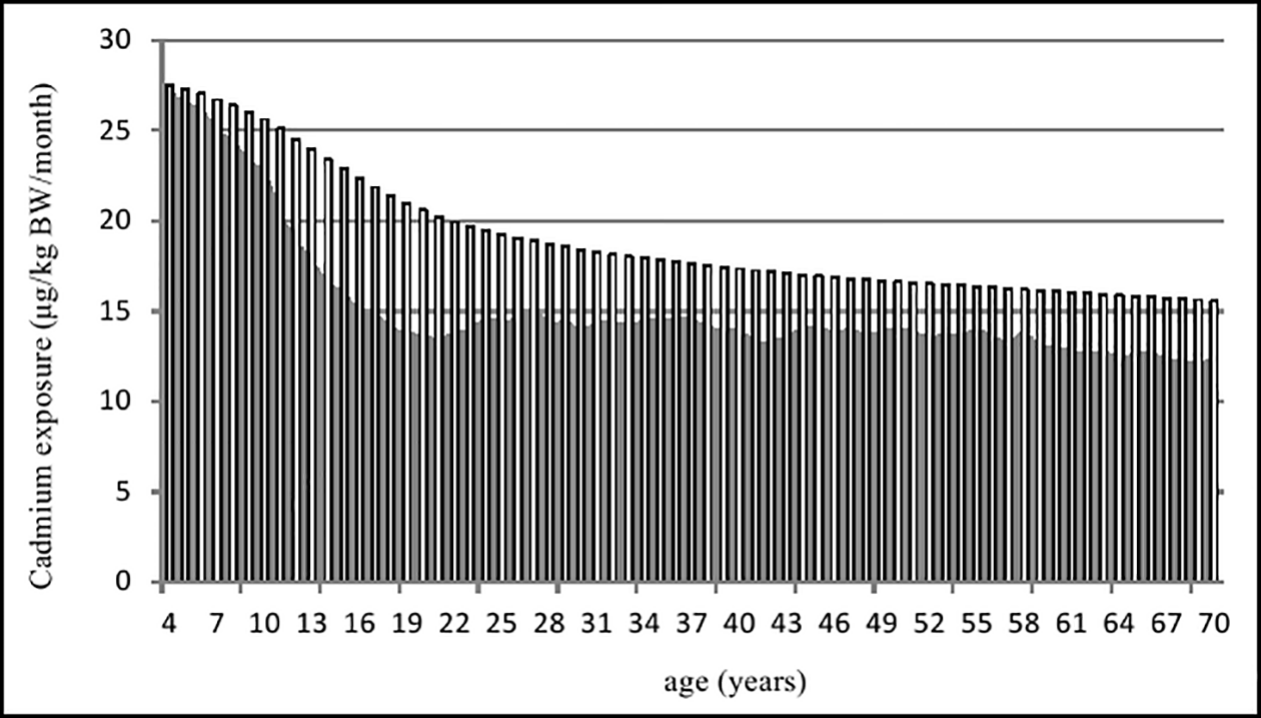
Trends of cadmium exposure with age. (black column: mean exposures of each age; white column: mean exposures of a period of time from 4 years to any age point).

However, the cadmium exposure of the Chinese population tended to decrease with age. Fig 1 shows the change of the mean dietary exposure of cadmium with age on the assumption that the consumption pattern and the cadmium concentration in foods would not change in the future. The mean exposure decreased rapidly with age until about 20 years old, then it remained relatively stable in adults (as the black column shown in Fig 1). In a long term view, the mean cadmium exposure per month for a period of time from 4 years old to any age point (such as 4–5 years old, 4–6 years old, etc) gradually decreased with the increase of chronic exposure duration (as the white column shown in Fig 1).

#### 2.2. Contributions of different food groups to cadmium exposure

**Fig 2:**
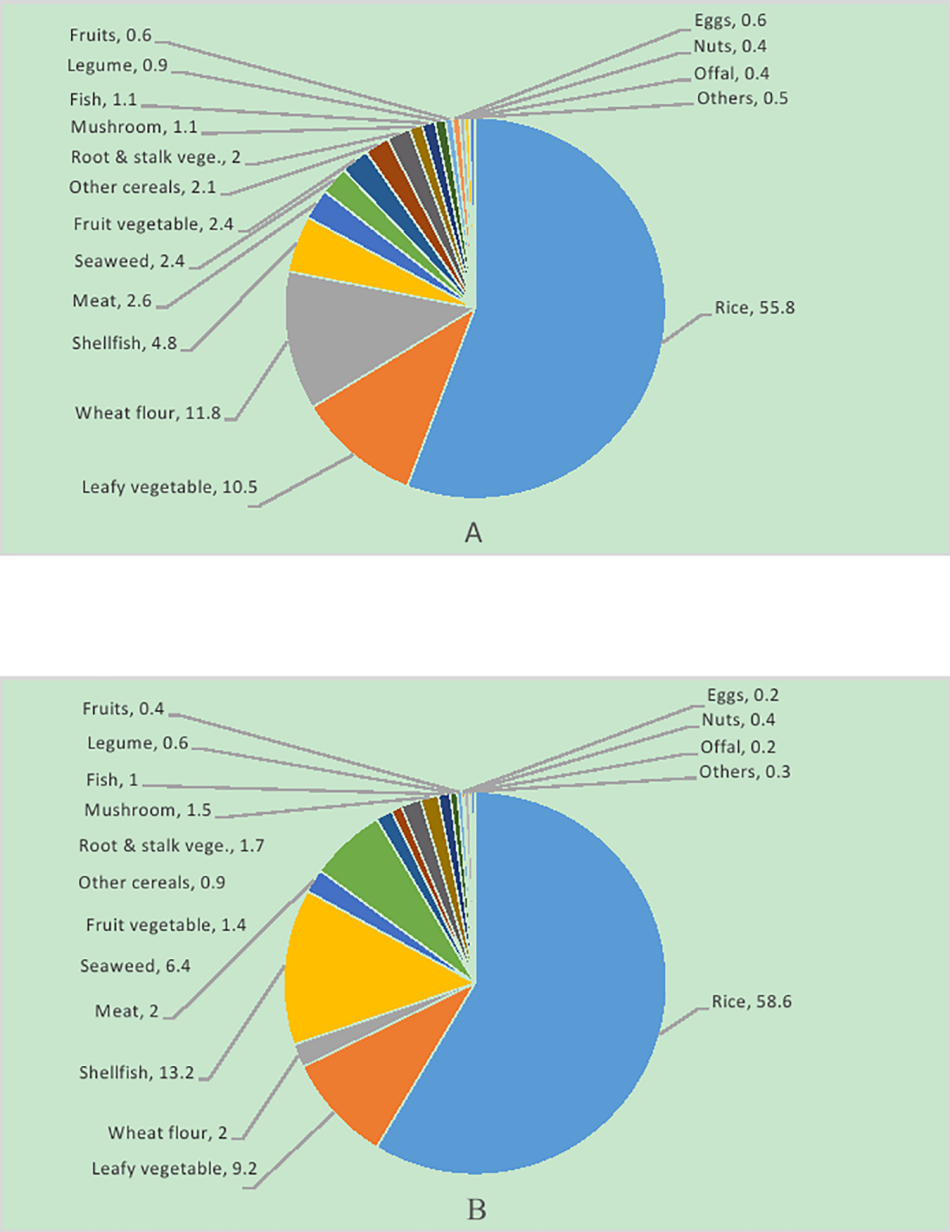
Contributions of dietary cadmium intake for Chinese population. (A, general population; B, high exposure population).

Each food group was separately analyzed for the general population and age-sex groups. Because there was no apparent difference in food contribution to cadmium exposure among age-sex groups, the contribution to cadmium exposure in the general population only is shown in Fig 2. The three most important contributors of dietary cadmium intake were rice, leafy vegetables and wheat flour. Because of its high levels of cadimumand consumption, rice contributed over 55.8% of the cadmium intake. Leafy vegetables and wheat flour contributed 11.8% and 10.5% of the cadmium intake, respectively, mostly due to their higher consumptions. Although the levels of cadmium in purple seaweed, sea crab, molluscs, mushroom, kelp and kidney were much higher, they contributed little to the total dietary exposure because of very small amounts of consumption.

For the high exposure sub-population with cadmium exposure higher than the 95^th^ percentile, rice was still the highest contributor (58.6%), followed by shellfish (13.2%) and leafy vegetables (9.2%). Comparing their contribution to that of the general population, wheat was no longer such an important contributor with a contribution of less than 2%, but the foods with high concentrations, such as seaweed, contributed more (6.4%).

#### 2.3. Comparison of the cadmium exposure between northern and southern population

The boundary of North and South in China is defined geographically by the Huai River and Qinling Mountains. There is a big difference on dietary pattern between northern and southern population in China, and therefore their dietary cadmium exposure was different. For both the general population and the high consumer group, the dietary cadmium exposure of the southern population was significently higher than that of the northern population (P<0.01) by a factor 1.7–1.8 for the general population and factors in the narrow range of 1.5–1.8 times for different age-sex groups (Table 3).

**Table 3:** Comparison of cadmium exposure between the northern and southern population.

age-sex group	Cadmium exposure (Mean±SD)		
	North (μg/kg BW/month)	South (μg/kg BW/month)	Factor between south and north
4-11yr	15.7±8.5	28.4±13.5	1.8*
12-17yr,male	12.9±7.2	22.3±10.7	1.7*
12-17yr,female	11.5±6.5	19.8±10.2	1.7*
≥18yr,male	10.1±5.7	17.1±8.4	1.7*
≥18yr,female	9.8±5.8	17.2±8.4	1.8*
Total population	11.0±6.9	19.5±11.0	1.8*

*: statistically significant different between the northern and southern population at p<0.01.

**Fig 3:**
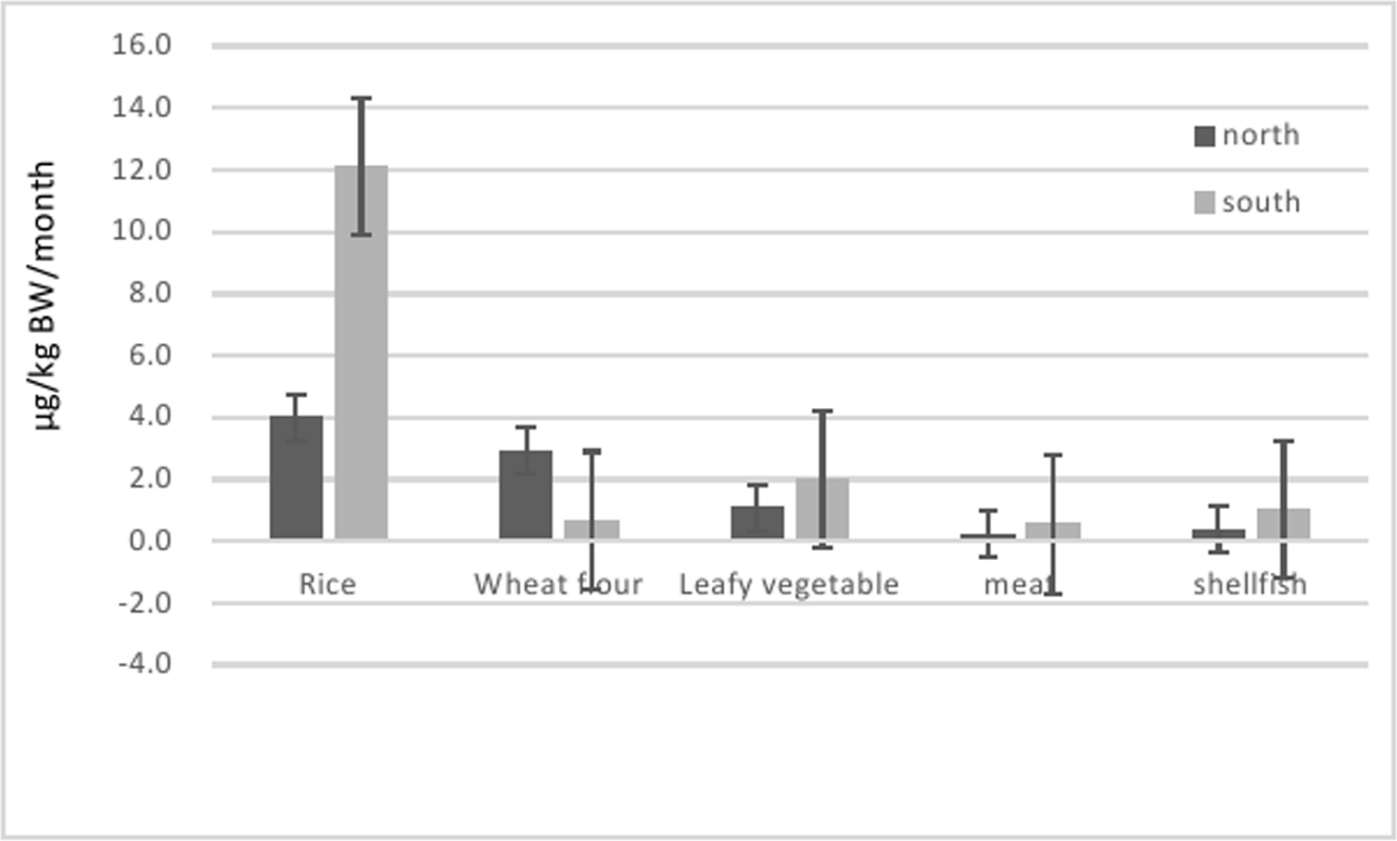
Estimated northern and southern consumer exposure to cadmium by different food groups.

When comparing the contribution of food groups to cadmium exposure of the general population, the southern population exposed more cadmium than the northern population from rice, leafy vegetables, meat and shellfish but not from wheat. Rice, as the most important contributor for both northern (37.8%) and southern populations (65.1%), and contributed as twice as much cadmium exposure to the southerner than that to the northerner (Fig 3). In contrast, the northern population exposed higher cadmium via wheat flour than did the southern population because of their higher wheat flour consumption.

## Discussion

The mean dietary cadmium exposure of the general Chinese population was 15.3 μg/kg BW/month (30.6 μg/day for a 60 kg BW of adults), which equated to 61.2% of the PTMI (25 μg/kg BW/month) established by JECFA. This value was higher than previous estimates in Chinese Total Diet Studies (13.8 μg/d, 19.4 μg/d and 22.2 μg/d in 1990, 1992 and 2000, respectively) [25–27], which indicated an increased trend of cadmium dietary exposure in China. Compared to reported values in other Asian countries, the cadmium exposure of Chinese adults was similar to that of Bangladeshis (34.6 μg/d) [29], but lower than that of male adults (50–56 μg/d) in the Bangkok area of Thailand [30], and higher than Japan (22.8 μg/d) [31], Lebanon (15.8μg/d) [32], and Republic of Korea (14.5 μg/d) [33]. The dietary cadmium exposures of countries outside Asia were usually very low. The cadmium exposure of the European population was 7.6 (6.4–9.6) μg/kg BW/month as estimated by EFSA, Australia with 2.2–6.9 μg/kg BW/month, United States with 4.6 μg/kg BW/month, Chile with 9 μg/kg BW/month as reported by JECFA [34,35].

Children (4–11 yr) exposed more cadmium than young people (12-17yr) and adults (over 18yrs), whilst not exceeding the PTMI. One possible reason for the higher intake in children was that food consumption as a factor of body weight (kg/BW) was greater in children than in other sub-group populations. Considering the cumulative toxicity and long half-life of cadmium, a long-term exposure assessment was more important in the risk assessment. Although people were exposed to more cadmium in the stages of early childhood, the cadmium exposure level per month tended to decrease with the increased age. A lifetime average exposure of 16.6 μg/kg BW/month was estimated for a person of 50 years old at which age steady cadmium contents in human kidneys were expected to be achieved [35]. This value was closed to the estimated exposure of the general population illuminated by this study.

The major dietary contributions of cadmium exposure were identified in many countries. The food groups that contributed to the most exposure among different countries over more than a decade were rice and grain, shellfish and sea food, meat including edible offal, and vegetables [36–41]. In USA, the top three major contributors were shellfish and food containing sea foods, wheat dishes and bread, and potatoes [37]. In Europe, grains, vegetables and meat contributed the most to the top exposure [39]. In this study, for children and adults, the top three contributors were rice, leafy vegetables and wheat flour. Rice was the most important contributor because of its higher consumption as a major food and higher cadmium concentration. According to some previous reports, rice was also the most important contributor of the dietary cadmium exposure in some countries such as Vietnam (90%), Korea (31%), and Thailand [36, 38, 40].

In this study, there was significant difference in cadmium exposure between southerners and northerners in China. The dietary cadmium exposure of the southern population was apparently higher than that of the northern population. The same conculsion was derived in a previous Chinese study in which the food consumption data source was the same as our study, but only 2629 food samples divided into 10 food categories were analyzed and the exposure estimate method was different. In that study, the results showed that the adults in the north of China had a mean cadmium intake of 2.23 μg/kg BW/week (9.56 μg/kg BW/month), while the southern population had a higher mean cadmium intake of 3.87 μg/kg BW/week (16.59 μg/kg BW/month) [42], which were slightly lower than those of our study. The difference in cadmium exposure between southerners and northerners was mostly due to the difference in consumption pattern. In China, southerners usually eat more rice, leafy vegetables and seafood than northerners do. Among the five highest food contributors to the mean cadmium exposure of the general population in China, southerners had higher intake of cadmium than northerners from rice, leafy vegetables, meat and shellfish except for wheat only. Especially for rice, as the most important contributor for the general population, although it was still the highest contributor for both the northerner (37.8%) and the southerner (65.1%), it contributed about twice as much cadmium exposure to southerners as for northerners. In contrast, cadmium exposed from wheat by the northerner was more than that by the southerner because of their higher wheat consumption.

There were some uncertainties in this study. For example, certain foods, such as beans, were not included in the analysis despite 32 categrories of main foods consumed by Chinese being surveyed. The absence of certain foods might result in the underestimation of the true exposure to cadmium via foods. In addition, the food consumption data were collected more than 10 years ago, and the changes in Chinese dietary pattern due to the rapid development of the economy in China were not taken into consideration [43]. All of these uncertainties might lead into underestimation of the exposure of the Chinese population. The higher undetectable rate of cadmium in some food items was another important factor influencing the results. When different strategies were applied to deal with the concentration data lower than LOD, the estimated results might be different. In this situation, the value of LOD was used. This strategy was adopted by most of studies to make relatively conservative assessment of risk (reasonable overestimate). Sometimes, both “0” and “LOD” were used to obtain an exposure range [44–46]. Moreover, only exposure through foods was calculated and this did not account for additional exposure pathways such as smoking and inhalation exposure which may also play important role for human cadmium burden [47, 48].

## Conclusion

In a long term view, the health risk from dietary cadmium exposure of the general Chinese population was low for mean consumers, but the risk for high consumers cannot be excluded. Rice was the main contributor. The health risk of cadmium exposure of certain sub-groups should be of concern.

## Supporting information

S1 File Food consumption. (XLSX)
